# The clinical outcomes of S-1 plus cisplatin for patients with advanced gastric cancer: A meta-analysis and systematic review

**DOI:** 10.1097/MD.0000000000012789

**Published:** 2018-12-10

**Authors:** Lei Yang, Xingcheng Wang, Binsheng Wang, Peng Chao, Debang Li, Chen Chai

**Affiliations:** a Department of General Surgery, First Hospital of Lanzhou University; b The First Clinical College of Lanzhou University, Lanzhou University, Lanzhou, Gansu, P.R. China.

**Keywords:** chemotherapy, cisplatin, gastric cancer, meta-analysis, S-1

## Abstract

**Background:**

To evaluate the clinical outcomes of S-1 plus cisplatin (SC) for the treatment of patients with advanced gastric cancer (AGC).

**Methods:**

A systematic literature search was conducted by searching PubMed, the Cochrane Library, Embase, China Biology Medicine disc (CBMdisc), China National Knowledge Infrastructure (CNKI), and WanFang Database, for all year up to January 2017. Pooled analyses of overall survival (OS), progress-free survival rates, and adverse events were performed.

**Results:**

A total of 8 random controlled trails (RCTs) consisting of 2699 patients with AGC were selected and included in this meta-analysis. The results of our meta-analysis showed that AGC patients who treated with SC regimen receive a similar OS (HR = 1.01, 95%CI: 0.86–1.18, *P* = .928), PFS (HR = 0.89, 95%CI: 0.72–1.09, *P* = .263), and overall response rate (HR = 0.88, 95%CI: 0.70–1.11, *P* = .283). However, SC regimen may increase the risk of 1 to 2 grade (OR = 1.128, 95%CI: 1.075–1.184, *P* = .000) and 3 to 4 grade (OR = 1.24, 95%CI: 1.01–1.52, *P* = .039) adverse events.

**Conclusion:**

SC chemotherapy showed no difference in survival compared with 5-FU- and S-1-based other therapy, but has a higher rate of adverse events compared with other chemotherapy regimens.

## 1 Introduction

Gastric cancer (GC), with an estimated 1.3 million new cases in 2015, is one of the most prevalent malignancies and the third-leading cause of cancer-related mortality in the world.^[1]^ Only approximately 25% of all patients with GC have resectable disease at presentation.^[2]^ However, there are regional differences, and especially GC is more common in East Asia than in western countries.^[3]^ Although surgery carries a high cure rate for stage IA and IB cancers, the results for stage IIIA and IIIB tumors are more poorer. In the western word, most patients are diagnosed in advanced stage, especially in IIIA/IIIB stage, which are technically inoperable.^[4]^ Results for both resectable and locally advanced gastric cancer (AGC) may be improved by either preoperative or adjuvant chemotherapy.^[5]^ Patients with inoperable, recurrent, or metastatic tumors have a poor prognosis with a median survival time of 3 to 5 months without chemotherapy.^[6]^

The standard treatment for AGC including both recurrent and metastatic diseases is palliative chemotherapy.^[7]^ Recently, increasing evidences have shown that the first-line chemotherapy resulted in a survival benefit relative to best supportive care.^[8]^ The most widely accepted first-line chemotherapy regimen is a combination of fluoropyrimidine (i.e., 5-fluorouracil, S-1, or capecitabine) plus platinum (i.e., oxaliplatin or cisplatin) with or without trastuzumab according to human epidermal growth factor receptor type 2 status.^[9]^ A randomized phase III trial of S-1 plus cisplatin (SC) versus S-1 In RCT In the Treatment for Stomach cancer (SPIRITS) showed the superiority of S-1 (an oral fluoropyrimidine-derivative dihydropyrimidine dehydrogenase inhibitor) plus cisplatin combination therapy (CS) to S-1 monotherapy in the first-line chemotherapy for Japanese patients with AGC.^[10]^ However, no high-quality meta-analysis has been conducted to assess the efficacy and safety of SC versus other chemotherapy regimens. Therefore, we performed a meta-analysis to evaluate the clinical outcomes of SC for the treatment of patients with AGC.

## 2 Materials and methods

Because this study is a meta-analysis of previously published studies, the ethical approval and patient consent are not required.

### 2.1 Literature search

Databases including PubMed, Embase, the Cochrane Library, China Biology Medicine disc (CBMdisc), China National Knowledge Infrastructure (CNKI), and WanFang Database were systematically searched from their inception to January 2017. The literature search was conducted by using MeSH terms, keywords, and combined words, which included Gastric cancer, Gastric Neoplasm, Gastric Carcinoma, Stomach Cancer, Stomach Neoplasm, Stomach Carcinoma, S-1, and gimeracil plus oteracil potassium plus tegafur. The meta-analysis was performed according to the Preferred Reporting Items for Systematic Reviews and Meta-analyses statement.

### 2.2 Inclusion criteria

Eligible studies for this meta-analysis met the following criteria: patients were confirmed to have AGC by pathological or histological examination; the studies comparing the clinical outcomes of S-1 combined with cisplatin and S-1 alone, cisplatin alone, or other treatments; and the studies reporting at least one point: overall survival (OS), progression-free survival (PFS) rates, and adverse events.

### 2.3 Exclusion criteria

The following studies were excluded from the meta-analysis: the original articles which did not report the comparative outcomes about the therapeutic value of sorafenib-RFA and other treatments; review articles, case reports, abstracts, editorials, letters, and meta-analysis; articles without sufficient data to analyze after contacting the authors of the study; and duplicate publications.

### 2.4 Data extraction

Two reviewers independently extracted relevant data from the included studies by using a predesigned data form. Any disagreements were resolved by discussion with other researchers. Data retrieved from each publication included basic characteristics of each study such as the first author, year of publication, country, sample size, chemotherapy regimens, research duration, and the time of follow-up and clinical outcomes such as OS, PFS, and adverse events.

### 2.5 Quality assessment

Quality assessment for each eligible study was carried out by the same 2 reviewers who independently read and scored each publication, according to the Cochrane risk of bias tool (Cochrane Handbook for Systematic Reviews of Interventions).^[11]^ When discrepancy occurred, a third author was referred. We rated the following domains separately for each of the included studies as “low risk of bias,” “high risk of bias,” and “unclear” when the risk of bias was uncertain or unknown: generation of allocation sequence; concealment of allocation; prevention of knowledge of the allocated interventions; methods used to address incomplete outcome data; selective outcome reporting; and other sources of bias that could put a study at high risk of bias, including whether a calculation of sample size was carried out including baseline comparability.

### 2.6 Statistical analysis

All statistical analyses were performed by using the software STATA 12.0. Heterogeneity was evaluated with a χ^2^-based Q-test: if the *P*-value was >.1 or *I*^2^ was <50%, it demonstrated that all included studies were lacking of heterogeneity, and the Mantel–Haenszel method (fixed effect model) was used to merge the studies. Otherwise the random effect model was adopted. Calculation for dichotomous variables was carried out using the odds ratio (OR) and their 95% confidence interval (95%CI) as the summary statistic. Two-sided *P* < .05 was considered statistically significant. Sensitivity analysis was performed to evaluate the stability of the results. Publication bias was evaluated by using the Begg test and Egger test.

## 3 Results

### 3.1 The characteristics of included studies

**Figure 1:**
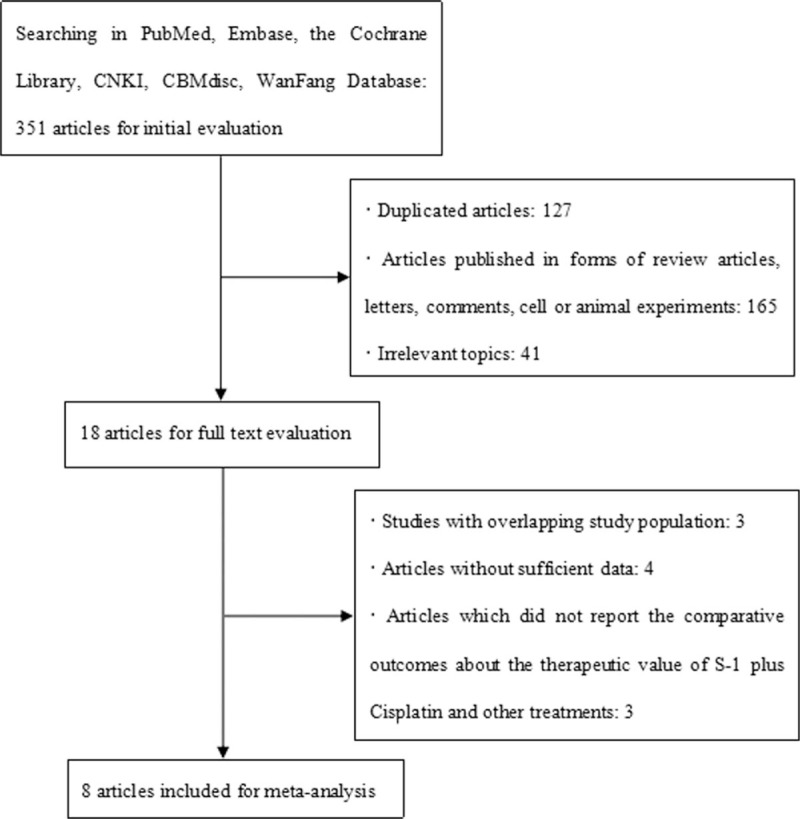
Flow chart of study selection process.

According to the search strategies in different databases, 351 unique references were identified through our searching the databases from their inception to January 2017, from which 127 were excluded by Endnote X6, and 206 were excluded after title and abstract screening, because of ineligibility according to the criteria for this meta-analysis. Of the 18 reports remaining for full-text reading, 3 studies were eligible to assess SC versus 5-Fu plus cisplatin, 1 study was eligible to assess SC versus cisplatin alone, 2 studies were eligible to assess SC versus S-1 combined with oxaliplatin or leucovorin, and 2 studies were eligible to assess SC versus S-1, cisplatin combined with other treatment (i.e., TSU68, leucovorin). Following the inclusion and exclusion criteria, 8 RCTs^[8,11–17]^ consisting of 2699 AGC patients were selected and included in this meta-analysis. Detailed information about the flow chart of study selection process is reported in Figure 1.

**Figure 2:**
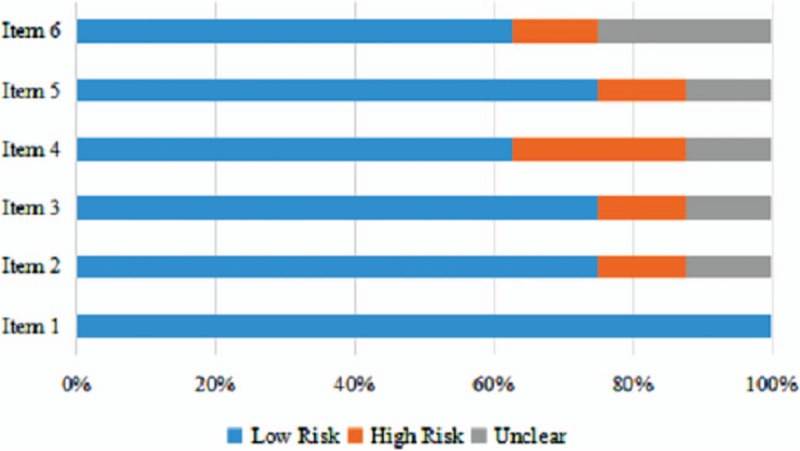
The quality assessment of included studies by Cochrane risk of bias tool. Item1-6: (1) generation of allocation sequence; (2) concealment of allocation; (3) prevention of knowledge of the allocated interventions; (4) methods used to address incomplete outcome data; (5) selective outcome reporting; and (6) other sources of bias that could put a study at high risk of bias, including whether a calculation of sample size was carried out including baseline comparability.

There are no major differences in studies and patient characteristics among the included studies. The baseline characteristics are shown in Table 1. For the quality assessment of the included studies, all studies reported the generation of allocation sequence; 1 study (12.5%, 1/8) existed high risk in concealment of allocation and revention of knowledge of the allocated interventions; 2 studies (25.0%, 2/8) existed high risk in methods used to address incomplete outcome data; and 75% of all studies showed low risk in selective outcome reporting; 2 studies (25.0%, 2/8) did not report the details about other sources of bias (Fig. 2).

**Table 1:**
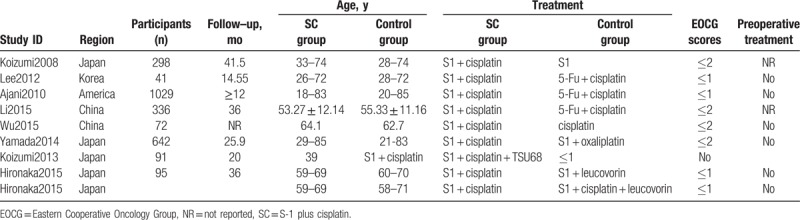
The baseline characteristics of included studies.

### 3.2 Meta-analysis of overall survival

**Figure 3:**
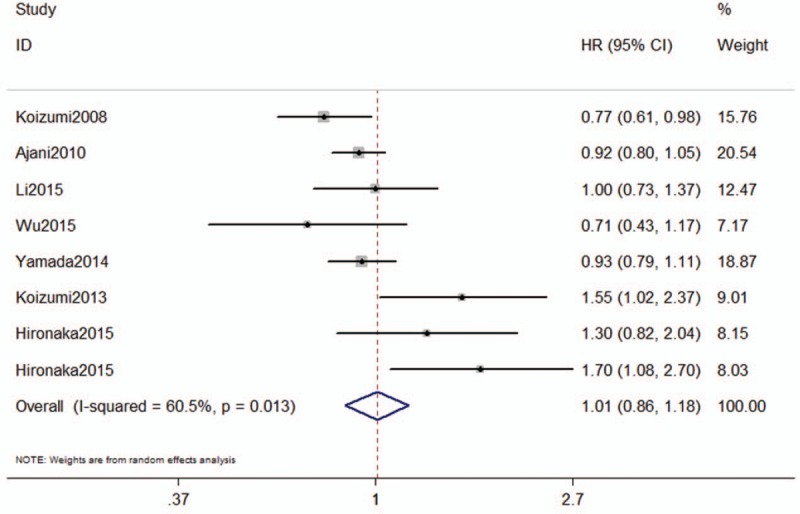
The meta-analysis results of overall survival for patients with advanced gastric cancer (AGC).

Seven of the included studies reported the results of OS for patients with AGC who were treated with SC. With observable interstudy heterogeneity (*I*^2^ = 60.5, *P* = .013), there are no significant difference in OS (HR = 1.01, 95%CI: 0.86–1.18, *P* = .928) (Fig. 3).

However, compared with S-1 monotherapy, SC regimen showed significant difference in OS (HR = 0.77, 95%CI: 0.61–0.98, *P* = .031). There is a worse OS (HR = 1.61, 95%CI: 1.18–2.20, *P* = .003) for AGC patients who treated with SC, compared with S-1, cisplatin combination with TSU68 or leucovorin. There were no statistically difference in cisplatin alone (HR = 0.71, 95%CI: 0.43–1.17, *P* = .180), 5-Fu plus cisplatin (HR = 0.93, 95%CI: 0.82–1.06, *P* = .270), and S-1 plus oxaliplatin or leucovorin (HR = 1.03, 95%CI: 0.77–1.38, *P* = .860).

### 3.3 Meta-analysis of progression-free survival

**Figure 4:**
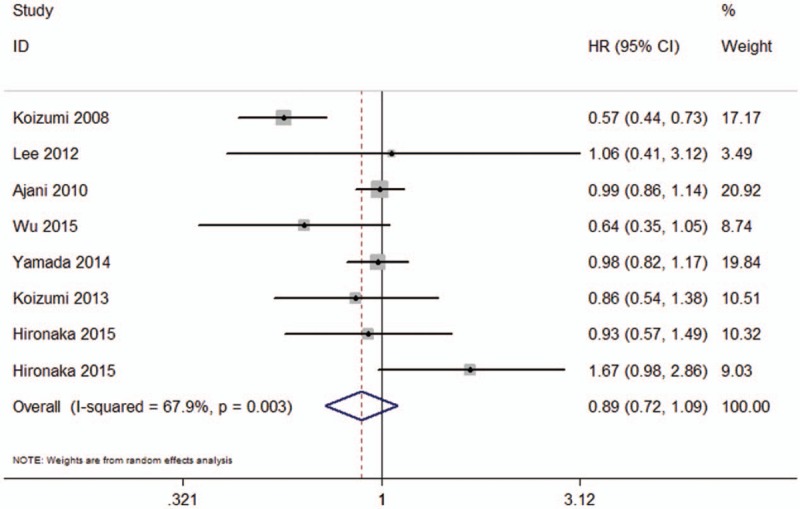
The meta-analysis results of progression-free survival for patients with advanced gastric cancer (AGC).

There are 7 RCTs which reported the results of PFS for patients with AGC. Heterogeneity existed between studies (*I*^2^ = 67.9%, *P* = .003). The random effects model was used to perform the meta-analysis. However, there is no significant difference between SC and other chemotherapy regimens in PFS (HR = 0.89, 95%CI: 0.72–1.09, *P* = .263) (Fig. 4).

Compared with S-1 alone chemotherapy, SC regimen showed a more favorable PFS (HR = 0.57, 95%CI: 0.44–0.73, *P* = .001). Meanwhile, there were no significant differences in cisplatin alone (HR = 0.64, 95%CI: 0.37–1.11, *P* = .902), 5-FU plus cisplatin (HR = 0.99, 95%CI: 0.86–1.14, *P* = .111), S-1 combined with oxaliplatin or leucovorin (HR = 0.97, 95%CI: 0.82–1.15, *P* = .740), and S-1, cisplatin combined with other treatment (HR = 1.18, 95%CI: 0.62–2.26, *P* = .613).

### 3.4 Meta-analysis of overall response rate

**Figure 5:**
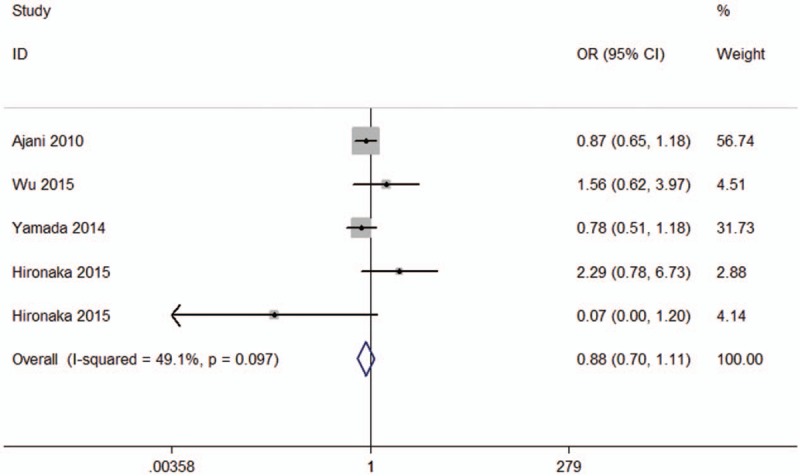
The meta-analysis results of overall response rate for patients with advanced gastric cancer (AGC).

We also conducted a meta-analysis to evaluate the overall response rate between SC and other chemotherapy regimens. Interstudy heterogeneity did not exist (*I*^2^ = 49.1%, *P* = .097). Meta-analysis showed that there is no significant difference between SC and other chemotherapy regimens (HR = 0.88, 95%CI: 0.70–1.11, *P* = .283) (Fig. 5).

### 3.5 Meta-analysis of 1 to 2 grade adverse events

All included studies reported the overall adverse events. The results of meta-analysis were showed that the rate of 1 to 2 grade adverse events (OR = 1.128, 95%CI: 1.075–1.184, *P* = .000) was increased for patients treated with SC. Compared to other chemotherapy regimens, there are significant difference in anemia (OR = 1.891, 95%CI: 1.587–2.250, *P* = .000), anorexia (OR = 1.293, 95%CI: 1.085–1.540, *P* = .004), nausea (OR = 1.204, 95%CI: 1.027–1.413, *P* = .022), fatigue (OR = 1.203, 95%CI: 1.023–1.414, *P* = .026), stomatitis (OR = 0.681, 95%CI: 0.566–0.819, *P* = .000), and increased creatinine (OR = 2.955, 95%CI: 2.299–3.797, *P* = .000) (Table 2).

**Table 2:**
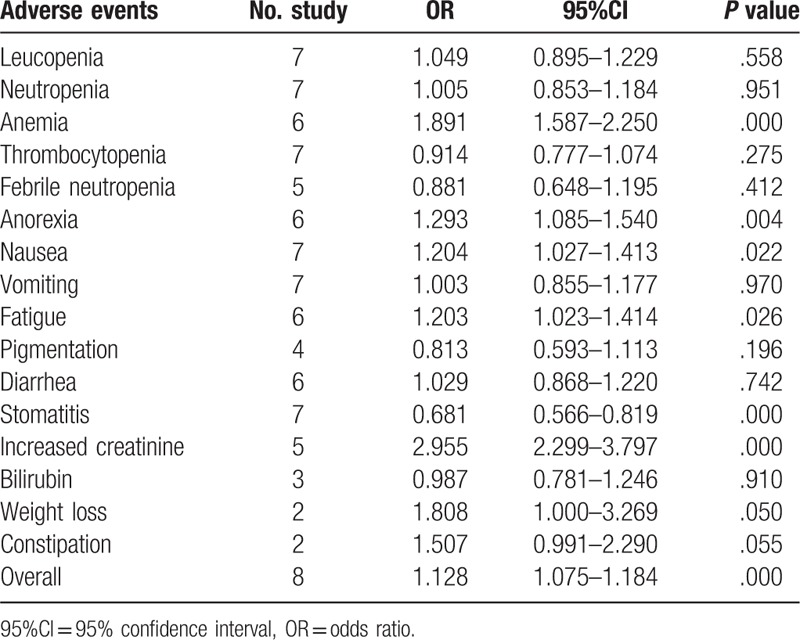
Meta-analysis of 1 to 2 grade adverse events.

### 3.6 Meta-analysis of 3 to 4 grade adverse events

**Figure 6:**
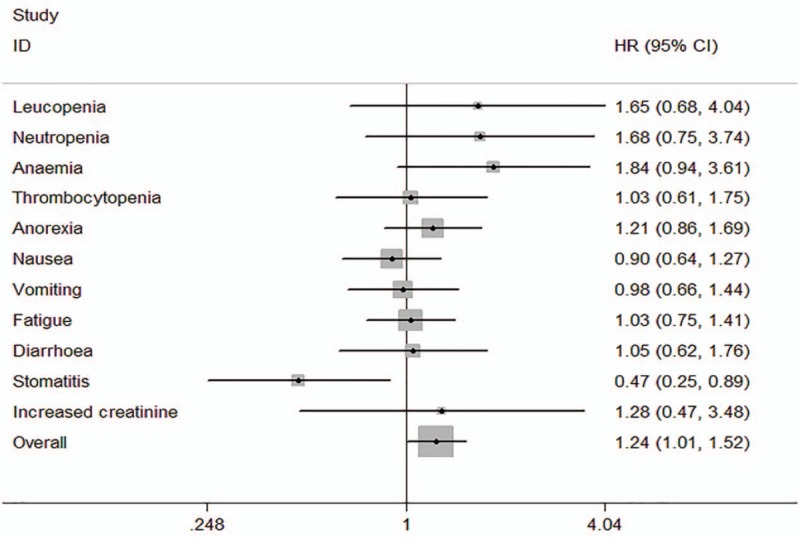
Meta-analysis of 1 to 2 grade adverse events.

We performed a meta-analysis to evaluate grade 3 to 4 adverse events. The results are shown in Figure 5. Compared with other chemotherapy regimens, SC have a lower rate of stomatitis (OR = 0.47, 95%CI: 0.25–0.89, *P* = .020). However, with a significant difference, SC regimen increased the overall rate of grade 3 to 4 adverse events (OR = 1.24, 95%CI: 1.01–1.52, *P* = .039) (Fig. 6).

## 4 Discussion

There were approximately 951,600 new cases of GC documented and 723,100 know deaths caused by GC in 2012.^[18]^ As we all known, complete resection is essential for the cure of AGC; however, even after complete resection, tumor recurrence can develop.^[19]^ Surgical resection can remove the visible tumor tissue from the operative fields, but it cannot eradicate the micrometastatic tumor cells that exist outside of the surgical field. The aim of adjuvant therapy is to eradicate micrometastatic tumor cells before or after surgery to improve the patients’ survival.^[20]^ Because of a survival benefit in favor S-1, previous meta-analysis has suggested that 5-FU may be replaced by S-1 in the first-line chemotherapy for patients with AGC.^[21]^ S-1 was regarded to have advantages than capecitabine among Asians in terms of reducing incidence of toxicities.^[22]^ Several chemotherapeutic regimens combining S-1 with other anticancer agents have been shown to improve the response rate or median survival time in gastric cancer.^[23]^ From among many possible combinations of anticancer drugs, we conducted a meta-analysis to compare the efficacy and safety of SC with other chemotherapy regimens (i.e., S-1 alone, cisplatin alone, 5-FU plus cisplatin, S-1 plus oxaliplatin/leucovorin, S-1 combination with cisplatin and TSU68, and S-1 combination with cisplatin and leucovorin).

The results of our meta-analysis showed that AGC patients who treated with SC regimen receive a similar OS, PFS, and overall response rate. However, SC regimen may increase the risk of adverse events. For AGC patients, the use of combination chemotherapy could be considered as standard of care for first-line treatment.^[24]^ The results of this meta-analysis suggest a significant and conclusive survival benefit for SC versus S-1 or cisplatin alone. As many studies included in this comparison have used combination chemotherapy regimens with suboptimal efficacy, such as 5-FU/cisplatin, S-1/cisplatin/TSU68, the benefit of combination chemotherapy is likely to be underestimated. In the absence of contraindications, the upfront use of a 2-drug combination is efficacious. However, 3-drug combinations are not widely used in clinical practice, which might be beneficial for individual people.

In previous studies^[25]^ of S-1 containing treatment regimens for gastric cancer, the proportion of patients with a response was about 30% for S-1 monotherapy and 50% to 54% for S-1 plus cisplatin; median PFS was 4 months versus about 6 months; and OS was 11 months versus about 13 months.^[26]^ The overall response and PFS of patients in the S-1 plus leucovorin group were similar to those in patients given SC, which has been reported to be more effective than S-1 monotherapy.^[27]^ Trials in patients with metastatic colorectal cancer and pancreatic cancer have shown an enhancement of antitumor activity with S-1 plus leucovorin compared with S-1 alone.^[28,29]^ Moreover, the hazard ratio for OS was 0.77 for S-1 plus leucovorin versus SC although this was not a significant difference.^[30]^ OS might be affected by subsequent regimens of chemotherapy, which could alter the survival effect of first-line treatment.

Admittedly, there are several limitations in our meta-analysis. First of all, heterogeneity was remarkable in our meta-analysis, which might exist in the sample size, age, study region, and history of previous treatments of the patients. Second, we did not take specific dosing regimens into account, which could have impacted our results. With pooled data analyses, including meta-analysis, it is often not possible to investigate to what extent dose differences may have influenced the results of the meta-analysis. We hope that future randomized controlled studies may resolve this problem and provide us with much more sound clinical evidence.

In summary of current evidence, SC chemotherapy showed no difference in survival compared with 5-FU- and S-1-based other therapy, but has a higher rate of adverse events compared with other chemotherapy regimens.

## Author contributions

**Conceptualization:** Lei Yang, Binsheng Wang, Debang Li, Chen Chai.

**Data curation:** Lei Yang, Xingcheng Wang, Peng Chao, Debang Li.

**Funding acquisition:** Debang Li.

**Investigation:** Binsheng Wang.

**Methodology:** Xingcheng Wang, Binsheng Wang, Debang Li.

**Project administration:** Peng Chao, Debang Li, Chen Chai.

**Resources:** Lei Yang, Peng Chao, Debang Li, Chen Chai.

**Software:** Lei Yang, Peng Chao, Chen Chai.

**Supervision:** Lei Yang.

**Validation:** Binsheng Wang.

**Writing – original draft:** Lei Yang, Xingcheng Wang, Debang Li, Chen Chai.

**Writing – review & editing:** Lei Yang, Chen Chai.
